# Acquired Immunodeficiency Syndrome With Suspected Early Inflammatory Bowel Disease Diagnosed Following Gastrointestinal Symptoms

**DOI:** 10.7759/cureus.82600

**Published:** 2025-04-19

**Authors:** Tomiko Ryu, Tubasa Tateishi, Satoshi Saito, Shiho Iwamoto, Keiko Abe

**Affiliations:** 1 Department of Hematology, Tokyo Yamate Medical Center, Tokyo, JPN; 2 Department of Gastroenterology, Tokyo Yamate Medical Center, Tokyo, JPN; 3 Department of Pathology, Tokyo Yamate Medical Center, Tokyo, JPN

**Keywords:** colonoscopy, hiv infection, immune-mediated diseases, inflammatory bowel disease, pathological findings

## Abstract

A 35-year-old man who has sex with men (MSM) visited another hospital for soft stools, lower abdominal pain, and nausea. Gastroscopy (GS) and colonoscopy (CS) revealed esophageal candidiasis and rectal ulcers, for which fluconazole (FLCZ) and metronidazole (MNZ) were prescribed. Four days later, the patient was referred to our hospital. Blood tests showed human immunodeficiency virus (HIV) infection (CD4: 116/µL, HIV-1mRNA: 2.4 × 105 copies/mL). Computed tomography (CT) revealed rectal wall thickening and fluid retention. CS showed ulcers in the rectum, and pathological findings of ulcer margins showed disturbances in the running of the crypts and a decrease in goblet cells. Symptoms improved with fasting and fluid replacement.

After discharge, gastrointestinal symptoms flared up, and he was readmitted. CT revealed mild wall thickening and fluid retention in the entire colon. However, CS revealed that the rectal ulcers tended to regress. Since the clinical manifestations, CT findings, and CS findings were not consistent, biopsies were performed at nine random sites from the ileum to the rectum. Pathological findings showed inflammation in the entire colon, compatible with inflammatory bowel disease (IBD). The patient was diagnosed with early-stage IBD.

One month later, antiretroviral therapy (ART) was initiated. Three months later, CS revealed that the ulcers in the rectum were scarred, and pathological findings from the nine randomly biopsied sites showed disappearances of inflammation. In people living with HIV (PLWH) who develop gastrointestinal symptoms, IBD should be considered in differential diagnosis.

## Introduction

Human immunodeficiency virus (HIV) infection belongs to immune-mediated inflammatory diseases (IMIDs) and is associated with the development of other IMIDs, systemic lupus erythematosus, psoriasis, autoimmune thyroid diseases, and rheumatic diseases [1]. Inflammatory bowel disease (IBD) also belongs to IMIDs, and IMIDs associated with IBD include asthma, psoriasis, rheumatic diseases, multiple sclerosis, primary sclerosing cholangitis, and pyoderma gangrenosum [2,3]. However, the risk of developing IBD after HIV infection remains unknown.

In the narrow sense, IBD includes idiopathic ulcerative colitis (UC) and Crohn’s disease (CD), whereas, in the broad sense, IBD includes infectious enteritis caused by bacterial, parasitic, viral, or fungal infection; drug-induced enteritis; radiation-induced enteritis; ischemic enteritis; and obstructive enteritis [4].

IBD is increasing worldwide, and Asia is one of the regions with the highest rates. Advances in the diagnosis and treatment of IBD have improved patient prognosis. For further improvement, it is important to make an accurate early diagnosis and treatment [5]. In diagnosis, it is necessary to rule out infectious IBD, such as HIV infection, and to diagnose any other IMIDs. In treatment, it is important to provide comprehensive treatment that includes not only IBD but also any complications.

Patients with both HIV and IBD represent a rare patient population. In both diseases, it is suggested that chronic inflammation due to dysbiosis of the intestinal symbiotic microflora caused by dysfunctioning of the mucosal barrier, a decrease in the antimicrobial peptide α-defensin and structural changes, and changes at the cellular level are involved [6,7]. In humans, β-defensin, together with α-defensin, is expressed in a variety of tissues and exerts a broad impact on HIV-1 transmission, replication, and disease progression [8]. In humans, only α- and β-defensins are expressed in various tissues and have broad impacts on HIV-1 transmission, replication, and disease progression. 

It is important to consider the possibility of IBD in people living with HIV (PLWH) with gastrointestinal symptoms and to diagnose by CS and biopsy pathology, as well as to consider treatment, including antiretroviral therapy (ART). Herein, we present a case of acquired immunodeficiency syndrome (AIDS) with suspected early inflammatory bowel disease diagnosed following gastrointestinal symptoms.

## Case presentation

A 36-year-old man who has sex with men (MSM) began experiencing soft stools, lower abdominal pain, and nausea in December 2023 and visited another hospital in January 2024. He underwent gastroscopy (GS) and colonoscopy (CS), which revealed esophageal candidiasis and rectal ulcers, for which fluconazole (FLCZ) and metronidazole (MNZ) were prescribed. Due to the lack of improvement in symptoms, the patient was referred to our hospital four days later. The patient had no notable medical history. Physical examination revealed a body temperature (BT) of 36.8°C, no oral abnormalities, and clear breath sounds. Abdominal examination revealed a flat and soft abdomen with mild spontaneous pain and tenderness in the lower abdomen but no rebound tenderness, hepatosplenomegaly, palpable superficial lymph nodes, or abnormalities in the anal region. Laboratory findings revealed mild hepatic and renal dysfunction and electrolyte abnormalities. The HIV antigen/antibody (Ag/Ab) screening test was positive, and HIV-1 infection was diagnosed through confirmatory testing with HIV-1/2-specific antibodies. CD4 level was 116/μL, and HIV-1 mRNA level was 2.4 × 105 copies/mL. Syphilis infection was also confirmed (Table 1).

**Table 1:** Laboratory findings after the first and second hospitalization HIV: Human immunodeficiency virus, PT-INR: Prothrombin time-international normalized ratio, APTT: Activated partial thromboplastin time

	1st admission	2nd admission	Reference range
White blood cell (μL)	5460	7150	3500-9000
Neutrophil (%)	60.7	70.4	37.0-72.0
Lymphocyte (%)	27.3	22.4	19.0-49.0
Hemoglobin (g/dL)	15.1	15.1	14.0-18.0
Platelet (x104/μL)	21.7	26.7	12.0-36.0
PT-INR	1.5	―	0.85-1.15
APTT (sec)	33.4	ー	24-39
D-dimer (μg)	1.2	―	0.0-0.9
Albumin (g/dL)	3.6	2.8	3.9-4.9
Aspartate aminotransferase (U/L)	138	103	10-33
Alanine aminotransferase (U/L)	85	92	38-113
Blood urea nitrogen. (mg/dL)	17	39	9-20
Serum creatinine (mg/dL)	1.28	2.14	0.65-1.07
Serum sodium (mEq/L)	129	130	135-145
Serum potassium (mEq/L)	3.1	2.8	3.4-5.0
Serum chloride (mEq/L)	91	97	98-108
C-reactive protein (mg/dL)	0.6	0.3	0-0.4
HIV Antigen Antibody	(+)	―	(-)
HIV-1/2 specific antibodies	(+)	―	(-)
CD4 (/μL)	116	119	500-1000
HIV-viral load (copies/mL)	2.4x105	―	―
Hepatitis B surface antigen	(-)	―	(-)
Hepatitis B surface antibody	(+)	―	(-)
Hepatitis C virus antibody	(-)	―	(-)
Rapid plasma reagin (R.U.)	195.9	―	<1.0
Treponema pallidum antibody (T.U.)	522.4	ー	<10
Cytomegalovirus antigen (C7-HRP)	(-)	―	(-)

**Figure 1:**
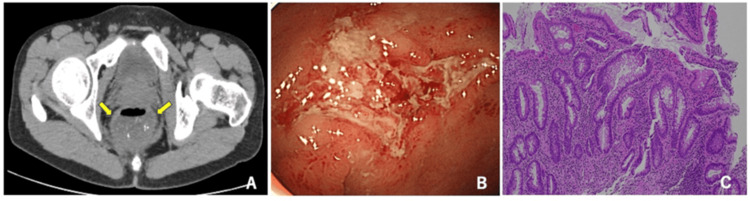
Findings of CT, CS and pathological findings after the first hospitalization A: CT findings revealing rectal wall thickening and fluid retention, B: CS findings revealing ulcers in the rectum and no other abnormalities, C: Pathological findings revealing disorganized running of the crypts and a decrease in goblet cells CT: computed tomography, CS: colonoscopy

CT on admission revealed circumferential wall thickening and internal fluid retention in the rectum (Figure 1A). CS revealed no abnormalities except for ulcers in the rectum (Figure 1B). Microscopic examination of intestinal fluid revealed no amebic trophozoites and intestinal fluid culture was negative for pathogenic bacteria and acid-fast bacteria. Rectal biopsy tissue was negative for periodic acid Schiff (PAS) staining, and no cytomegalovirus (CMV) giant cells were observed. Kaposi sarcoma and malignant lymphoma were also ruled out. Pathological findings of rectal ulcer margins showed disorganized running of the crypts and a decrease in goblet cells (Figure 1C). After hospitalization, symptoms improved with fasting and fluid replacement. Amoxicillin (AMPC) was initiated for syphilis infection, fluconazole (FLCZ) was resumed, and the patient was discharged after five days of hospitalization.

**Figure 2:**
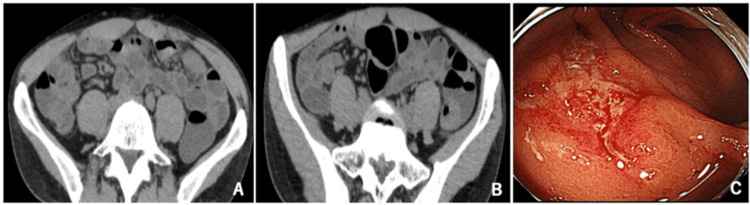
CT and CS findings after the second hospitalization A, B: CT findings showing mild wall thickening in the ileum, wall thickening in the entire colon, and fluid retention in the colon. C: CS findings revealed that the rectal ulcers tended to regress, and there was no obvious abnormality other than in the rectum.

One week after being discharged, the patient developed nausea and vomiting after meals, watery diarrhea with more than 10th lines per day, and persistent lower abdominal pain, which caused difficulties in walking; he visited our emergency room in late January. He was readmitted due to severe dehydration and renal dysfunction. CT on admission revealed mild wall thickening in the ileum, wall thickening in the entire colon, and fluid retention in the colon, which were consistent with diarrhea (Figures 2A, 2B). GS showed reflux esophagitis (Grade M), but esophageal candidiasis had disappeared. CS revealed that the rectal ulcers tended to regress, and there was no obvious abnormality other than in the rectum (Figure 2C).

**Figure 3:**
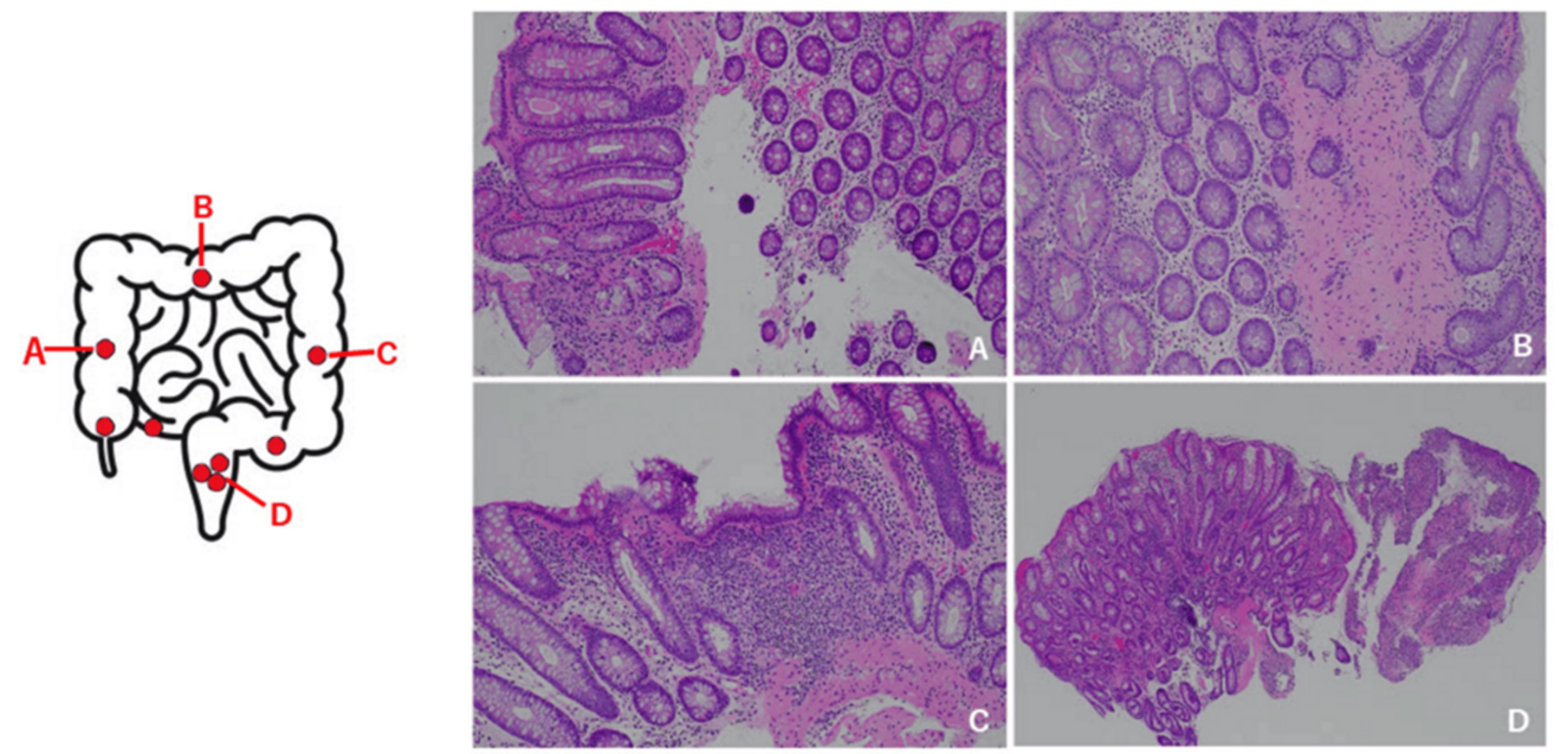
Biopsy pathological findings after the second hospitalization Left: Colon diagram (Olympus colorectal schema-default settings). The nine random biopsy sites are indicated with 🔴. Right: Biopsy pathological findings (from ascending colon to rectum). A: Ascending colon, B: Transverse colon, C: Descending colon, D: Rectum In all, cryptitis, crypt abscesses, disorganized running of the crypts, and infiltration of inflammatory cells in the entire colon, suggest early IBD. IBD: inflammatory bowel disease.

Since the clinical manifestations, CT findings, and CS findings were not consistent, nine biopsies were performed randomly from the terminal ileum to the rectum for a close examination (Figure 3 [left]). Pathological findings included cryptitis, crypt abscesses, disorganized running of the crypts, and infiltration of inflammatory cells in the entire colon, suggesting early IBD (Figures 3A-3D). 

**Figure 4:**
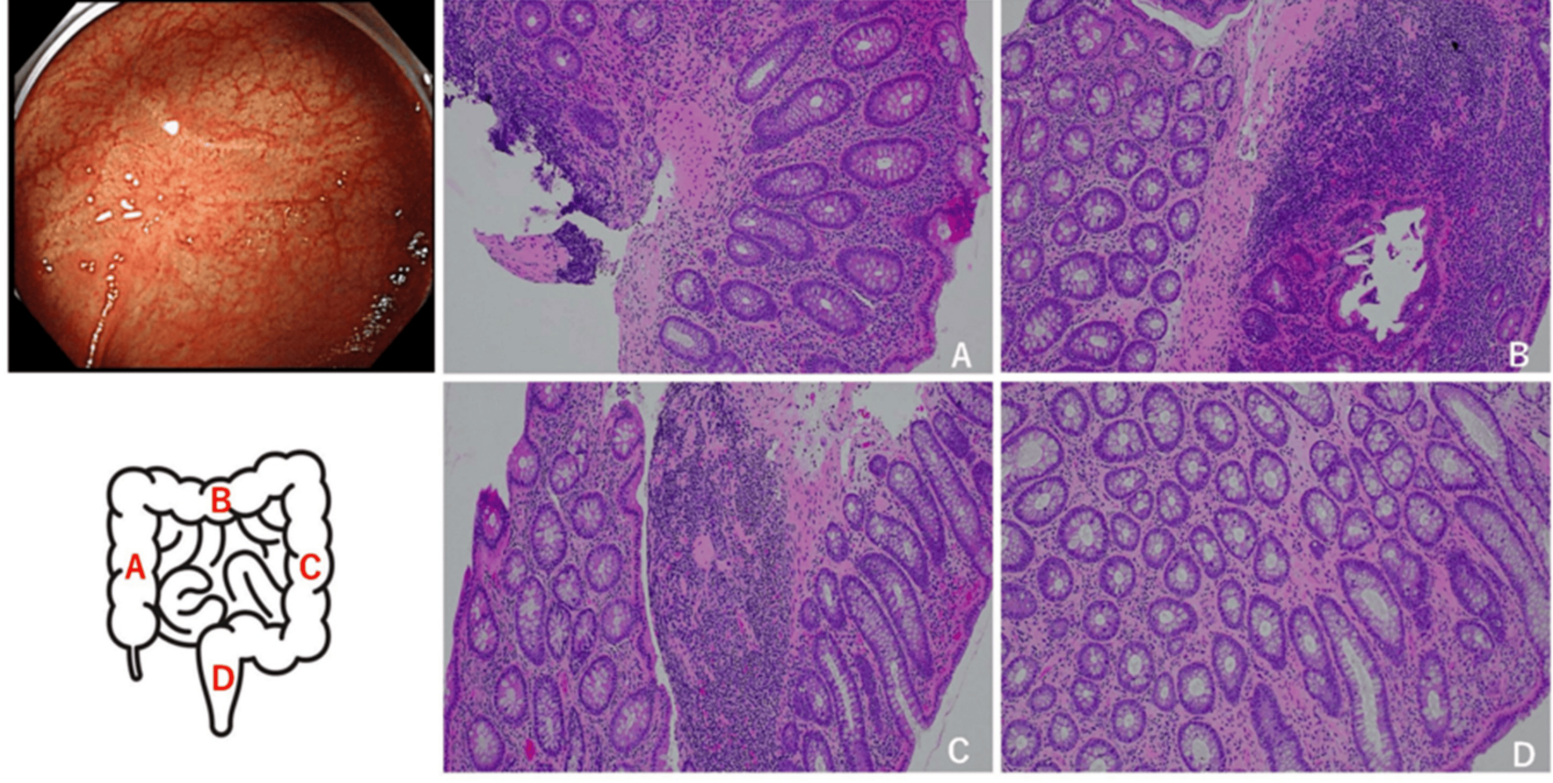
CS findings and biopsy pathological findings three months after discharge Upper left: CS findings Lower left: Colon diagram (Olympus colorectal schema-default settings). Right: Biopsy pathological finding. A: Ascending colon, B: Transverse colon, C: Descending colon, D: rectum. In all, signs of active inflammation had disappeared.

After admission, the patient was placed on fasting and fluid replacement. Diarrhea and lower abdominal pain improved after eight days. The antiretroviral therapy (ART) started in mid-February, then the patient was discharged. Three months after ART started, CD4 increased to 785/μL, and HIV-1 mRNA decreased to 5.4x10 copies/mL. CS was performed. The ulcers in the rectum were scarred (Figure 4 [upper left]), and pathology findings from the nine randomly biopsied sites showed that the signs of active inflammation had disappeared (Figures 4A-4D [lower left]). The patient’s condition improved without the administration of anti-inflammatory drugs, such as 5-ASA and steroid preparations, which are therapeutic agents for IBD. It has been more than one year since the patient was discharged, and no recurrence has been observed.

## Discussion

Although both HIV infection and IBD are IMIDs, the risk of IBD development in PLWH is not clear. In a Danish nationwide cohort study conducted among PLWH and sex- and age-matched HIV-negative individuals, Elmahdi et al. reported that the risk of IBD development in PLWH was more than twice as high as that in matched HIV-negative individuals. This risk was increased for all age groups and all calendar years of HIV diagnosis (including before and after ART). These findings were validated in a large cohort in the United States with high HIV prevalence [9]. The GETAID study is the first multicenter study to address the impact of HIV infection on the course of IBD and drug safety profile. In total, 195 patients with IBD, including 65 HIV-infected and 130 noninfected, were included after matching. In total, 38 patients (58.5%) were diagnosed with IBD after HIV infection. In disease type, for CD, colitis and perianal disease were significantly more prevalent in HIV-infected patients than in non-HIV-infected individuals, and for UC, proctitis was significantly more prevalent in HIV-infected patients than in non-HIV-infected individuals. The course of IBD in HIV-infected patients was not different from that in noninfected individuals for both CD and UC [10].

We experienced and reported a case of HIV infection complicated with UC at our hospital in 2006. The case was a 42-year-old man who had developed bloody stools and abdominal pain during the course of HIV infection (CD4: 350/μL, HIV-VL: 6 × 104 copies/mL). CS revealed continuous, and circumferential inflammation from the rectum to the cecum. Pathological findings showed disorganized running of the crypts, a decrease in goblet cells, and infiltration of inflammatory cells, and the patient was diagnosed with IBD/UC (severe pancolitis). Treatment was started with prednisolone (PSL) and salazosulfapyridine (SASP), after which the patient's symptoms improved, but it was discontinued due to the appearance of a drug-induced eruption. Subsequently, ART was initiated due to a decrease in CD4 count. One month later, the symptoms improved, and HIV-VL was below the detection limit. Approximately 10 months later, CS and pathological findings showed remission, and there has been no relapse until now, approximately 18 years later [11].

In the two cases encountered at our hospital, disease activity was monitored over time based on clinical, endoscopic, and pathological findings. With ART alone as a treatment, the patients achieved remission relatively early with no recurrence. It was suggested that the therapeutic effect may have been due to the suppression of immune response by the HIV virus. However, there have been no reports, as far as we could find in the literature, of HIV-VL in blood and intestinal mucosa before and after the initiation of ART.

In PLWH who develop lower gastrointestinal symptoms, IBD should be kept in mind as a differential diagnosis, including sexually transmitted diseases, viral colitis, and malignant diseases. In cases where a definitive diagnosis of IBD in the broad sense is made based on CS and pathological findings, early initiation of ART seemed to be useful if ART has not already been provided. In addition, MSM patients with lower gastrointestinal symptoms should be tested for sexually transmitted diseases, such as HIV and syphilis, and closely examined with IBD in mind. ART has been suggested to be effective as a treatment for IBD preceded by HIV infection in our experience.

## Conclusions

HIV infection and IBD are both IMIDs and associated with the development of other IMIDs, but not enough is known about the risk of IBD development in PLWH, its treatment, and the effectiveness of ART. Based on our experience with two cases of IBD in PLWH, we think that in PLWH, presenting with lower gastrointestinal symptoms, sexually transmitted diseases, and IBD in the broad sense, although rare, should be considered as a differential diagnosis, and diagnosis is necessary to be made based on CS and biopsy pathology for early diagnosis and treatment. In addition, in MSM patients with lower gastrointestinal symptoms, close examination should be performed with sexually transmitted diseases, such as HIV and syphilis, as well as IBD in mind. Furthermore, testing for sexually transmitted diseases is recommended in IBD patients with inflammation of the rectum/colon. A thorough understanding of differential diagnosis enables early diagnosis and treatment and is expected to improve prognosis. In the treatment of HIV infection preceding broadly defined IBD, ART might be useful if ART has not already been provided.
